# Evaluation of the relationship between dry eye syndrome severity and corneal sensitivity

**DOI:** 10.5935/0004-2749.2024-0202

**Published:** 2025-06-24

**Authors:** Zeynep Akgun, Pelin Kiyat, Idris Sarikaya, Ugur Yilmaz, Ozlem Barut Selver

**Affiliations:** 1 Department of Ophthalmology, Ege University Faculty of Medicine, Izmir, Turkey; 2 Department of Ophthalmology, Buca Seyfi Demirsoy Training and Research Hospital, Izmir Democracy University, Izmir, Turkey; 3 Department of Ophthalmology, Pamukkale University Faculty of Medicine, Denizli, Turkey

**Keywords:** dry eye disease, signs and symptoms, cornea, neuralgia, Cochet-Bonnet esthesiometer, sensory thresholds, surveys and questionnaires

## Abstract

**Purpose:**

This study aimed to evaluate the relationship between the objective severity of dry eye disease subjective symptoms, and corneal sensitivity.

**Methods:**

The study included 62 eyes from 31 healthy volunteers and 150 eyes from 75 patients diagnosed with dry eye disease . Participants underwent the Schirmer I test, tear break-up time assessment, and corneal staining evaluation using the Oxford Scale. Subjective symptoms were assessed through the Ocular Surface Disease Index questionnaire and a modified Ocular Surface Pain Score questionnaire. Corneal sensitivity was measured in five corneal regions using a Cochet-Bonnet esthesiometer. Dry eye disease severity was graded from 1 to 5 based on the Oxford Scale. Comparative analyses were performed.

**Results:**

Schirmer I and tear break-up time values were significantly lower in the DED group, while Ocular Surface Disease Index and Ocular Surface Pain Score were significantly higher (p<0.001 for all). Corneal sensitivity in all quadrants was significantly lower in DED patients (p<0.001 for all). A strong correlation was observed between the Ocular Surface Pain Score and the Ocular Surface Disease Index (r=0.983, p<0.001). Central corneal sensitivity exhibited a moderate positive correlation with Schirmer I and tear break-up time (p<0.001, r=0.583 and 0.657, respectively) and a moderate negative correlation with Ocular Surface Disease Index and Ocular Surface Pain Score (p<0.001, r=0.625 and -0.631, respectively). Disease severity progression was associated with increased Ocular Surface Disease Index and Ocular Surface Pain Score, but no statistically significant difference was found between Grades 3 and 5. Similarly, corneal sensitivity decreased with advancing disease severity, yet no significant difference was observed between Grades 4 and 5.

**Conclusion:**

Corneal sensitivity decreases in dry eye disease and is negatively correlated with disease severity. Subjective symptoms increase with disease progression and show a positive correlation with severity. The absence of significant differences between the advanced stages suggests that neuropathic mechanisms and subbasal nerve plexus deterioration play a role in chronic and late-stage dry eye disease.

## INTRODUCTION

Dry eye is a prevalent ocular surface disorder resulting from increased tear evaporation, reduced tear production, or a combination of both, with reported prevalence rates ranging from 5% to 34%^(1)^. According to the 2007 Dry Eye Workshop diagnostic guidelines, subjective symptoms play a crucial role in diagnosing dry eye disease (DED) alongside objective measures such as tear break-up time (TBUT), Schirmer tests, meibomian gland dysfunction assessment, tear osmolarity testing, and ocular surface staining^(2^,^3)^.

Dry eye disease is an inflammatory condition that shares characteristics with autoimmune diseases. Environ-mental, endogenous, genetic, and infectious factors are believed to disrupt ocular surface homeostasis, triggering disease onset^(4)^. Corneal nerves play a fundamental role in maintaining ocular surface integrity, and corneal nerve dysfunction is known to contribute to DED pathophysiology^(5^-^7)^. While it is well established that disrupted homeostasis and secondary corneal epithelial damage can lead to nerve impairment, neuropathic changes may occur even in the absence of epithelial pathology. Increased levels of proinflammatory cytokines, chemokines, and matrix metalloproteinases may exacerbate epitheliopathy by activating autoreactive Th1 and Th17 CD4+ T cells in the ocular surface and lacrimal gland^(8^,^9)^. Additionally, complement activation and CD4+ T cells have been implicated in corneal nerve damage independent of epithelial pathology in various animal models^(9^,^10)^. Although the exact mechanisms remain unclear, neuropathy and epitheliopathy appear to form a vicious cycle that disrupts ocular surface homeostasis, leading to somatosensory changes and negatively impacting quality of life.

Studies on corneal sensitivity in DED have yielded conflicting results, with reports of hypoesthesia, hyperesthesia, or no significant sensitivity changes. Patients with varying degrees of disease severity often report nociceptive, neuropathic, or psychogenic ocular pain^(11^-^14)^.

In routine clinical practice, ocular surface staining remains a critical tool for assessing epitheliopathy in DED. The Oxford Scale, which evaluates the pattern and intensity of staining, is particularly useful for grading disease severity and monitoring treatment responses, as it provides more comprehensive insights compared with numerical measurements alone^(15)^.

Given these considerations, this study aimed to evaluate the relationship between DED severity-graded using the Oxford Scale-subjective neuropathic symptoms, and corneal sensitivity.

## METHODS

This cross-sectional study included 62 eyes from 31 healthy volunteers who presented to the outpatient clinic for routine ophthalmological examination and 150 eyes from 75 newly diagnosed patients with DED who had not yet initiated treatment. The exclusion criteria were as follows: presence of any ocular disease other than refractive error or DED, use of contact lenses, chronic use of ocular or systemic medications, and history of ocular surgery.

Following a comprehensive ophthalmological examination, participants underwent the Schirmer I test, TBUT measurement, corneal staining assessment using the Oxford Scale, and administration of the Ocular Surface Disease Index (OSDI) and Ocular Surface Pain Score (OSPS) questionnaires. Corneal sensitivity was evaluated using a Cochet-Bonnet esthesiometer.

The Ocular Surface Disease Index (OSDI; Allergan, Inc., Irvine, California) is a validated questionnaire designed to assess the severity and impact of ocular surface symptoms associated with chronic DED. It evaluates three domains: ocular symptoms, visual function, and environmental triggers, making it a widely used tool in DED diagnosis, follow-up, and clinical research^(16^,^17)^. To focus on corneal pain sensitivity, the OSPS questionnaire was derived by modifying the OSDI, specifically by removing questions related to visual function (e.g., blurred vision and poor vision). Before undergoing clinical evaluations, all participants completed both the OSDI and OSPS.

After a routine slit-lamp examination, the Schirmer I test was performed without topical anesthesia using a standardized filter strip (5 × 35 mm) placed at the lower eyelid margin. The length of wetting on the strip was recorded after 5 min. TBUT was assessed by applying a fluorescein strip to the inferior fornix, and the time until the first break in the fluorescein tear film was recorded under cobalt blue filter slit-lamp examination.

Corneal and conjunctival staining patterns were evaluated using the Oxford Scale and graded from 0 to 5 by two independent masked clinicians. Corneal sensitivity was measured in five regions (central, superior, inferior, nasal, and temporal) using a Cochet-Bonnet esthesiometer (Luneau Technology, France) without topical anesthesia. Measurements were performed with the filament initially extended to 60 mm and progressively shortened until a positive response was elicited. Each measurement was repeated three times per region, and the mean value was recorded. To minimize variability, all assessments were conducted by the same clinician between 10:00 and 15:00 at a controlled room temperature.

According to the diagnostic guidelines established by Tear Film and Ocular Surface Dry Eye Workshop II (TFOS DEWS II), the Asian Dry Eye Society, and the Japanese Dry Eye Society, patients were classified as having DED if they met the following criteria: positive symptoms on the OSDI questionnaire (score ≥13) and at least one sign of ocular surface instability, including TBUT ≤10 s, Schirmer I test ≤10 mm, corneal staining (>5 corneal spots), conjunctival staining (>9 conjunctival spots), and lid margin staining^(18^,^19)^. Patients diagnosed with DED were categorized into five severity grades based on the Oxford Scale. Corneal sensitivity, dry eye parameters, OSDI, and OSPS scores were compared across severity groups and between patients with DED and healthy controls.

All statistical analyses were performed using IBM SPSS Statistics 25.0 (IBM Corp., Armonk, New York) and R 4.3.0 software. Numerical variables were expressed as mean, standard deviation, median, minimum, and maximum values. Chi-square tests were used to compare gender distributions between groups. Welch’s ANOVA was used to compare OSDI and OSPS scores across severity groups, with Bonferroni correction applied for pairwise comparisons. Since both eyes of each patient were analyzed, linear mixed-effects models were created for TBUT, Schirmer I test results, and corneal sensitivity measurements, with eye laterality (“side” variable), included as a random effect. Model suitability was tested using the restricted maximum likelihood (REML) method, and the Tukey correction was applied for *post hoc* pairwise comparisons. Pearson correlation analysis was performed for parametric variables, whereas Spearman correlation analysis was used for nonparametric variables. P-values were derived from *t* values and standard errors, with statistical significance set at p<0.05. Results were reported at a 95% confidence interval. This study was conducted in compliance with the Declaration of Helsinki and was approved by the Ege University Faculty of Medicine Ethics Committee (Approval No. 22-6T/39). The study received financial support from the Ege University Scientific Research Projects Coordination Office (Project ID: 27393). Written informed consent was obtained from all participants.

## RESULTS

The mean age of patients with DED was 48.17 ± 17.68 yr (range: 20-83), while the mean age of healthy volunteers was 44.35 ± 14.90 yr (range: 24-82), with no statistically significant difference between groups (p=0.162). The male-to-female ratio was 32:43 in the DED group and 19:12 in the control group, with a significantly higher female prevalence among patients with DED (p=0.014).

In alignment with previous studies, Schirmer I test and TBUT values were significantly lower, while OSDI and OSPS scores were significantly higher in the DED group than the control group (p<0.001 for all comparisons). Corneal sensitivity was significantly reduced across all quadrants in patients with DED (p<0.001 for all comparisons). Additionally, a strong positive correlation was observed between OSPS and OSDI scores (r=0.983, p<0.001).

When patients were stratified into five severity groups based on DED progression, mean age, and gender distribution are presented in table 1. Female predominance was particularly significant in Groups 3 and 4. The mean Schirmer I test, TBUT, OSDI, OSPS, and central corneal sensitivity values for both eyes are summarized in table 2.

**Table 1:** Demographic characteristics of the study groups

Groups	Mean age (yr) ± SD (Range)	F/M (n)
Healthy volunteers	45.96 ± 15.10 (24-82)	19/12
Grade 1 DED	47.40 ± 16.98 (21-82)	9/6
Grade 2 DED	46.43 ± 16.59 (24-69)	9/6
Grade 3 DED	48.83 ± 18.04 (21-83)	4/11
Grade 4 DED	48.76 ± 21.18 (20-83)	3/12
Grade 5 DED	48.33 ± 20.21 (20-82)	7/8

DED= dry eye disease.

**Table 2:** Schirmer I, TBUT, OSDI, OSPS, and central corneal sensitivity across study groups

Parameter	Groups					
	Healthy Volunteers	Grade 1	Grade 2	Grade 3	Grade 4	Grade 5
OSDI	6.81 ± 6.36	18.0 ± 14.34	49.3 ± 25.85	67.6 ± 16.69	66.66 ± 22.46	73.01 ± 14.03
OSPS	6.63 ± 6.22	19.96 ± 16.80	52.15 ± 25.73	73.65 ± 17.84	74.11 ± 20.97	82.76 ± 13.26
Schirmer I						
Right	21.29 ± 5.90	21.26 ± 6.13	14.86 ± 5.68	13.13 ± 6.63	13.66 ± 7.22	10.13 ± 5.31
Left	21.29 ± 5.91	21.13 ± 5.82	14.66 ± 7.72	12.46 ± 5.80	13.13 ± 7.70	10.00 ± 5.34
TBUT						
Right	15.29 ± 3.82	12.80 ± 3.72	8.60 ± 4.22	6.06 ± 2.25	4.40 ± 1.95	3.3 ± 1.54
Left	15.77 ± 3.72	13.13 ± 3.60	7.93 ± 4.04	5.46 ± 2.19	4.13 ± 1.59	3.66 ± 1.54
Central corneal sensitivity						
Right	59.35 ± 1.37	59.06 ± 2.60	56.80 ± 4.07	54.46 ± 5.55	49.20 ± 8.16	48.20 ± 10.01
Left	59.22 ± 1.82	59.06 ± 2.59	56.60 ± 3.90	54.26 ± 5.47	48.66 ± 8.83	47.66 ± 10.39

R-Right= L-Left, TBUT= tear break-up time, OSDI= Ocular Surface Disease Index, OSPS= Ocular Surface Pain Score.

A moderate positive correlation was observed between corneal sensitivity across all quadrants and both Schirmer I test results (r=0.583, p<0.001) and TBUT values (r=0.657, p<0.001). Conversely, a moderate negative correlation was found between corneal sensitivity and OSDI (r=-0.625, p<0.001) and OSPS (r=-0.631, p<0.001; Table 3).

**Table 3:** Correlation coefficients between corneal sensitivity in all quadrants and Schirmer I, TBUT, OSDI, and OSPS

Corneal sensitivities	Schirmer I	TBUT	OSDI	OSPS
Central	0.583	0.687	-0.625	-0.631
Superior	0.541	0.549	-0.556	-0.562
Inferior	0.548	0.564	-0.554	-0.565
Nasal	0.594	0.623	-0.608	-0.613
Temporal	0.561	0.598	-0.591	-0.593

* p<0.001 for all parameters, T-BUT= tear break-up time, OSDI= Ocular Surface Disease Index; OSPS= Ocular Surface Pain Score.

*Note:* p<0.001 for all parameters.

**Figure 1:**
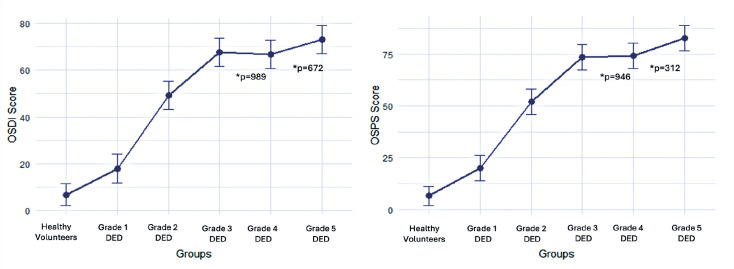
Pairwise comparisons of OSDI and OSPS scores among study groups

**Figure 2:**
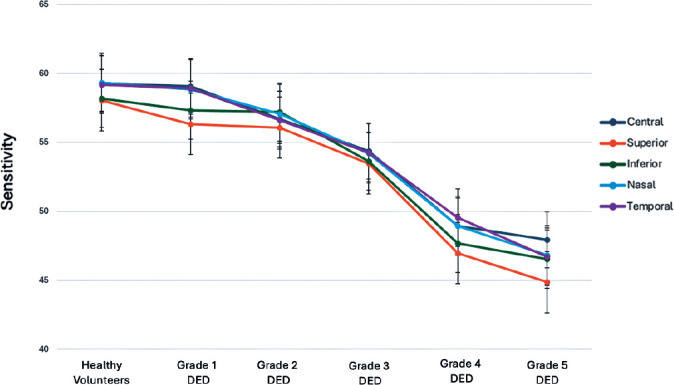
Pairwise comparisons of corneal sensitivity among study groups.

When comparing patients by DED severity, OSDI and OSPS scores increased with disease progression; however, there was no statistically significant difference in pairwise comparisons Grades 3 and 5 (Figure 1). Similarly, corneal sensitivity decreased as the disease progressed, with the most significant drop occurring between Grades 3 and 4, while no significant difference was observed between Grades 4 and 5 (Figure 2).

Statistical significance values for comparisons:

Grade 3 versus Grade 4: Central, superior, inferior, nasal, and temporal corneal sensitivity: p=0.003, 0.002, 0.001, 0.008, and 0.037, respectively.

Grade 4 versus Grade 5: Central, superior, inferior, nasal, and temporal corneal sensitivity: p=0.983, 0.817, 0.972, 0.731, and 0.477, respectively.

## DISCUSSION

The literature presents conflicting findings regarding alternations in corneal sensitivity in DED. Spierer et al. ^(20)^ reported that increased dry eye symptoms and ocular pain result in corneal hypersensitivity. Similarly, Kaido et al.^(21)^ found heightened corneal sensitivity in patients with low TBUT. Situ et al.^(11)^ also observed significantly higher conjunctival and corneal sensitivities in the DED group compared with controls, as measured by a pneumatic Belmonte esthesiometer.

Conversely, Rahman et al.^(22)^, using the Cochet--Bonnet aesthesiometer, evaluated corneal sensitivity in 10 healthy individuals and 33 patients with DED and found reduced corneal sensitivity in the latter, particularly in those with aqueous insufficiency. They attributed this reduction to increased eye irritation, tear instability, ocular surface disease, and decreased blink rate. Likewise, Bourcier et al.^(13)^ used a Belmonte noncontact gas esthesiometer and reported decreased corneal sensitivity to mechanical, thermal, and chemical stimuli in patients with DED compared with controls.

Adatia et al.^(23)^ investigated the correlation between corneal sensitivity, subjective dry eye symptoms, and corneal staining in patients with Sjogren’s syndrome. They found that sensitivity declined as ocular surface disease severity increased. However, they also noted that subjective symptoms decreased in advanced DED, despite more severe objective signs. They proposed that the chronicity and severity of DED influence its clinical presentation, but they did not provide a clear pathophysiological explanation for this phenomenon. Further research, particularly using confocal microscopy, could elucidate these mechanisms. Benítez del Castillo et al.^(24)^ examined corneal sensitivity using the Cochet-Bonnet esthesiometer and assessed subbasal nerve density via *in vivo* confocal microscopy, finding reductions in both in DED patients. Similarly, Labbé et al.^(25)^ reported decreased corneal sensitivity and subbasal nerve density in DED, with a positive correlation between corneal nerve density and sensitivity. Additionally, studies in animal models have suggested that complement and CD4+ T cell-mediated neuropathy may contribute to these findings^(8^-^10)^. Future clinical research is needed to further clarify this mechanism.

In the present study, corneal sensitivity was significantly lower in the DED group than in healthy controls, and sensitivity further declined as disease severity increased. However, no statistically significant increase in OSDI and OSPS was observed beyond Grade 3, and the reduction in corneal sensitivity was not statistically significant beyond Grade 4.

Changes in ocular surface sensitivity in DED should be examined in the context of both nociceptive and neuropathic mechanisms^(26)^. A discrepancy often exists between objective indicators of tear dysfunction and patient-reported symptoms, likely due to neuropathic contributions to ocular sensitivity. Additionally, systemic diseases such as diabetes and Sjogren’s syndrome may exacerbate this mismatch^(27)^. Another key factor is corneal nerve plexus deterioration caused by DED. Broadly speaking, patients whose symptoms exceed their clinical signs may experience predominant neuropathic mechanisms, whereas those with more pronounced clinical signs may exhibit neurotrophic changes, as demonstrated *in vivo* confocal microscopy studies^(28^,^29)^. The lack of a statistically significant difference in corneal sensitivity reduction and symptom severity in advanced DED stages suggests the increasing role of neuropathic mechanisms in disease progression.

Several grading systems have been proposed for DED, incorporating subjective symptoms and objective parameters such as corneal and conjunctival staining, TBUT, Schirmer’s test results, tear meniscus height, osmolarity, and meibomian gland dysfunction^(2^,^30^,^31)^. Corneal staining plays a particularly important role in disease grading, with multiple scales available, including the National Eye Institute (NEI)/Industry scale, the Oxford scale, and the Sjögren’s International Collaborative Clinical Alliance Ocular Staining Score^(32^-^34)^. However, no consensus has been reached regarding a standardized grading system. Some recent studies have proposed deep learning-based grading systems^(35)^. In this study, patients were classified using the Oxford scale, and no significant differences were observed between the most severe stages (Grades 4 and 5). This finding suggests that these stages could potentially be combined into a single category in future classification models.

A notable aspect of this study is its use of a new questionnaire specifically designed to assess pain sensitivity in DED. Several existing questionnaires, including the five-item Dry Eye Questionnaire (DEQ-5), OSDI, and the Standard Patient Evaluation of Eye Dryness questionnaire, are commonly used to evaluate DED symptoms^(36^-^38)^. However, these instruments incorporate both visual and ocular surface symptoms, potentially confounding results. In this study, the OSPS questionnaire-a modified version of the OSDI that excludes visual comfort-related questions-was employed. The strong correlation between OSPS and OSDI scores suggests that OSPS may be a valuable tool for evaluating pain sensitivity in DED.

This study demonstrated that corneal sensitivity decreases in DED and is negatively correlated with disease severity, whereas subjective symptoms increase and are positively correlated with disease progression. The lack of statistically significant differences in symptom severity and corneal sensitivity reduction between the most advanced disease stages suggests a role for neuropathic mechanisms and subbasal nerve plexus deterioration in chronic and severe DED. Further molecular-level studies are needed to clarify these associations. A key limitation of this study is the absence of an assessment of participants’ conjunctival or general pain thresholds.

## References

[1] Stapleton F, Alves M, Bunya VY, Jalbert I, Lekhanont K, Malet F (2017). TFOS DEWS II Epidemiology Report. Ocul Surf.

[2] Lemp MA, Baudouin C, Baum J (2007). The definition and classification of dry eye disease: report of
the Definition and Classification Subcommittee of the International Dry Eye
WorkShop. Ocul Surf.

[3] Lin H, Yiu SC. (2014). Dry eye disease: A review of diagnostic approaches and
treatments. Saudi J Ophthalmol.

[4] Messmer EM. (2015). The pathophysiology, diagnosis, and treatment of dry eye
disease. Dtsch Arztebl Int.

[5] Pizzano M, Vereertbrugghen A, Cernutto A, Sabbione F, Keitelman IA, Shiromizu CM (2024). Transient receptor potential vanilloid-1 channels facilitate
axonal degeneration of corneal sensory nerves in dry eye. Am J Pathol.

[6] Guzmán M, Miglio M, Keitelman I, Shiromizu CM, Sabbione F, Fuentes F (2020). Transient tear hyperosmolarity disrupts the neuroimmune
homeostasis of the ocular surface and facilitates dry eye
onset. Immunology.

[7] Vereertbrugghen A, Galletti JG. (2022). Corneal nerves and their role in dry eye
pathophysiology. Exp Eye Res.

[8] Vereertbrugghen A, Pizzano M, Sabbione F, Keitelman IA, Shiromizu CM, Aguilar DV (2023). An ocular Th1 immune response promotes corneal nerve damage
independently of the development of corneal epitheliopathy. J Neuroinflammation.

[9] Royer DJ, Echegaray-Mendez J, Lin L, Gmyrek GB, Mathew R, Saban DR (2019). Complement and CD4+ T cells drive context-specific corneal
sensory neuropathy. eLife.

[10] Vereertbrugghen A, Pizzano M, Cernutto A, Sabbione F, Keitelman IA, Aguilar DV (2024). CD4+ T cells drive corneal nerve damage but not epitheliopathy in
an acute aqueous-deficient dry eye model. Proc Natl Acad Sci USA.

[11] Situ P, Simpson TL, Jones LW, Fonn D. (2008). Conjunctival and corneal hyperesthesia in subjects with dryness
symptoms. Optom Vis Sci.

[12] De Paiva CS, Pflugfelder SC. (2004). Corneal epitheliopathy of dry eye induces hyperesthesia to
mechanical air jet stimulation. Am J Ophthalmol.

[13] Bourcier T, Acosta MC, Borderie V, Borrás F, Gallar J, Bury T (2005). Decreased corneal sensitivity in patients with dry
eye. Invest Ophthalmol Vis Sci.

[14] Benítez-Del-Castillo JM, Acosta MC, Wassfi MA, Díaz-Valle D, Gegúndez JA, Fernandez C (2007). Relation between corneal innervation with confocal microscopy and
corneal sensitivity with noncontact esthesiometry in patients with dry
eye. Invest Ophthalmol Vis Sci.

[15] Srinivas SP, Rao SK. (2023). Ocular surface staining: current concepts and
techniques. Indian J Ophthalmol.

[16] Dougherty BE, Nichols JJ, Nichols KK. (2011). Rasch analysis of the Ocular Surface Disease Index
(OSDI). Invest Ophthalmol Vis Sci.

[17] Miller KL, Walt JG, Mink DR, Satram-Hoang S, Wilson SE, Perry HD (2010). Minimal clinically important difference for the ocular surface
disease index. Arch Ophthalmol.

[18] Shimazaki J. (2018). Definition and diagnostic criteria of dry eye disease: historical
overview and future directions. Invest Ophthalmol Vis Sci.

[19] Tsubota K, Yokoi N, Shimazaki J, Watanabe H, Dogru M, Yamada M, Asia Dry Eye Society (2017). New Perspectives on Dry Eye Definition and Diagnosis: A Consensus
Report by the Asia Dry Eye Society. Ocul Surf.

[20] Spierer O, Felix ER, McClellan AL, Parel JM, Gonzalez A, Feuer WJ (2016). Corneal mechanical thresholds negatively associate with dry eye
and ocular pain symptoms. Invest Ophthalmol Vis Sci.

[21] Kaido M, Kawashima M, Ishida R, Tsubota K. (2016). Relationship of corneal pain sensitivity with dry eye symptoms in
dry eye with short tear break-up time. Invest Ophthalmol Vis Sci.

[22] Rahman EZ, Lam PK, Chu CK, Moore Q, Pflugfelder SC. (2015). Corneal sensitivity in tear dysfunction and its correlation with
clinical parameters and blink rate. Am J Ophthalmol.

[23] Adatia FA, Michaeli-Cohen A, Naor J, Caffery B, Bookman A, Slomovic A. (2004). Correlation between corneal sensitivity, subjective dry eye
symptoms and corneal staining in Sjögren’s syndrome. Can J Ophthalmol.

[24] Benítez del Castillo JM, Wasfy MA, Fernandez C, Garcia-Sanchez J. (2004). An in vivo confocal masked study on corneal epithelium and
subbasal nerves in patients with dry eye. Invest Ophthalmol Vis Sci.

[25] Labbé A, Alalwani H, Van Went C, Brasnu E, Georgescu D, Baudouin C. (2012). The relationship between subbasal nerve morphology and corneal
sensation in ocular surface disease. Invest Ophthalmol Vis Sci.

[26] Sanchez V, Cohen NK, Felix E, Galor A. (2023). Factors affecting the prevalence, severity, and characteristics
of ocular surface pain. Expert Rev Ophthalmol.

[27] Vehof J, Sillevis Smitt-Kamminga N, Nibourg SA, Hammond CJ. (2017). Predictors of discordance between symptoms and signs in dry eye
disease. Ophthalmology.

[28] Ong ES, Felix ER, Levitt RC, Feuer WJ, Sarantopoulos CD, Galor A. (2018). Epidemiology of discordance between symptoms and signs of dry
eye. Br J Ophthalmol.

[29] Lee Y, Kim M, Galor A. (2022). Beyond dry eye: how co-morbidities influence disease phenotype in
dry eye disease. Clin Exp Optom.

[30] Baudouin C, Aragona P, Van Setten G, Rolando M, Irkeç M, Benítez del Castillo J, ODISSEY European Consensus Group members (2014). Diagnosing the severity of dry eye: a clear and practical
algorithm. Br J Ophthalmol.

[31] Murube J, Németh J, Höh H, Kaynak-Hekimhan P, Horwath-Winter J, Agarwal A (2005). The triple classification of dry eye for practical clinical
use. Eur J Ophthalmol.

[32] Bron AJ, Evans VE, Smith JA. (2003). Grading of corneal and conjunctival staining in the context of
other dry eye tests. Cornea.

[33] Whitcher JP, Shiboski CH, Shiboski SC, Heidenreich AM, Kitagawa K, Zhang S, Sjögren’s International Collaborative Clinical Alliance
Research Groups (2010). A simplified quantitative method for assessing
keratoconjunctivitis sicca from the Sjögren’s Syndrome International
Registry. Am J Ophthalmol.

[34] Feng J, Ren ZK, Wang KN, Guo H, Hao YR, Shu YC (2023). An automated grading system based on topological features for the
evaluation of corneal fluorescein staining in dry eye
disease. Diagnostics (Basel).

[35] Kim S, Park D, Shin Y, Kim MK, Jeon HS, Kim YG (2024). Deep learning-based fully automated grading system for dry eye
disease severity. PLoS One.

[36] Ngo W, Situ P, Keir N, Korb D, Blackie C, Simpson T. (2013). Psychometric properties and validation of the Standard Patient
Evaluation of Eye Dryness questionnaire. Cornea.

[37] Schiffman RM, Christianson MD, Jacobsen G, Hirsch JD, Reis BL. (2000). Reliability and validity of the Ocular Surface Disease
Index. Arch Ophthalmol.

[38] Chalmers RL, Begley CG, Caffery B. (2010). Validation of the 5-Item Dry Eye Questionnaire (DEQ-5):
discrimination across self-assessed severity and aqueous tear deficient dry
eye diagnoses. Cont Lens Anterior Eye.

